# Supplementary material for: DNA methylation either antagonizes or promotes Polycomb recruitment at transposable elements in Arabidopsis
Source: Nat Commun. 2026 Sep 2;17:10439. doi: 10.1038/s41467-026-77108-9 (PMC13631316; doi:10.1038/s41467-026-77108-9)
Supplement: Supplementary file 1 [file 41467_2026_77108_MOESM1_ESM.pdf]

DNA methylation either antagonizes or promotes Polycomb recruitment at transposable elements in Arabidopsis

Valentin Hure[1 †], Maghnia Kawter Elmir[1 †], Florence Piron-Prunier[1], Raphaël Guérois[1], Angélique Déléris[1]*

[1] Université Paris-Saclay, Commissariat à l'Energie Atomique (CEA), Centre National de la Recherche Scientifique (CNRS), Institute for Integrative Biology of the Cell (I2BC), 91190 Gif-sur-Yvette, France

[†] Contributed equally , * Correspondence to: angelique.deleris@i2bc.paris-saclay.fr

SUPPLEMENTARY INFORMATION

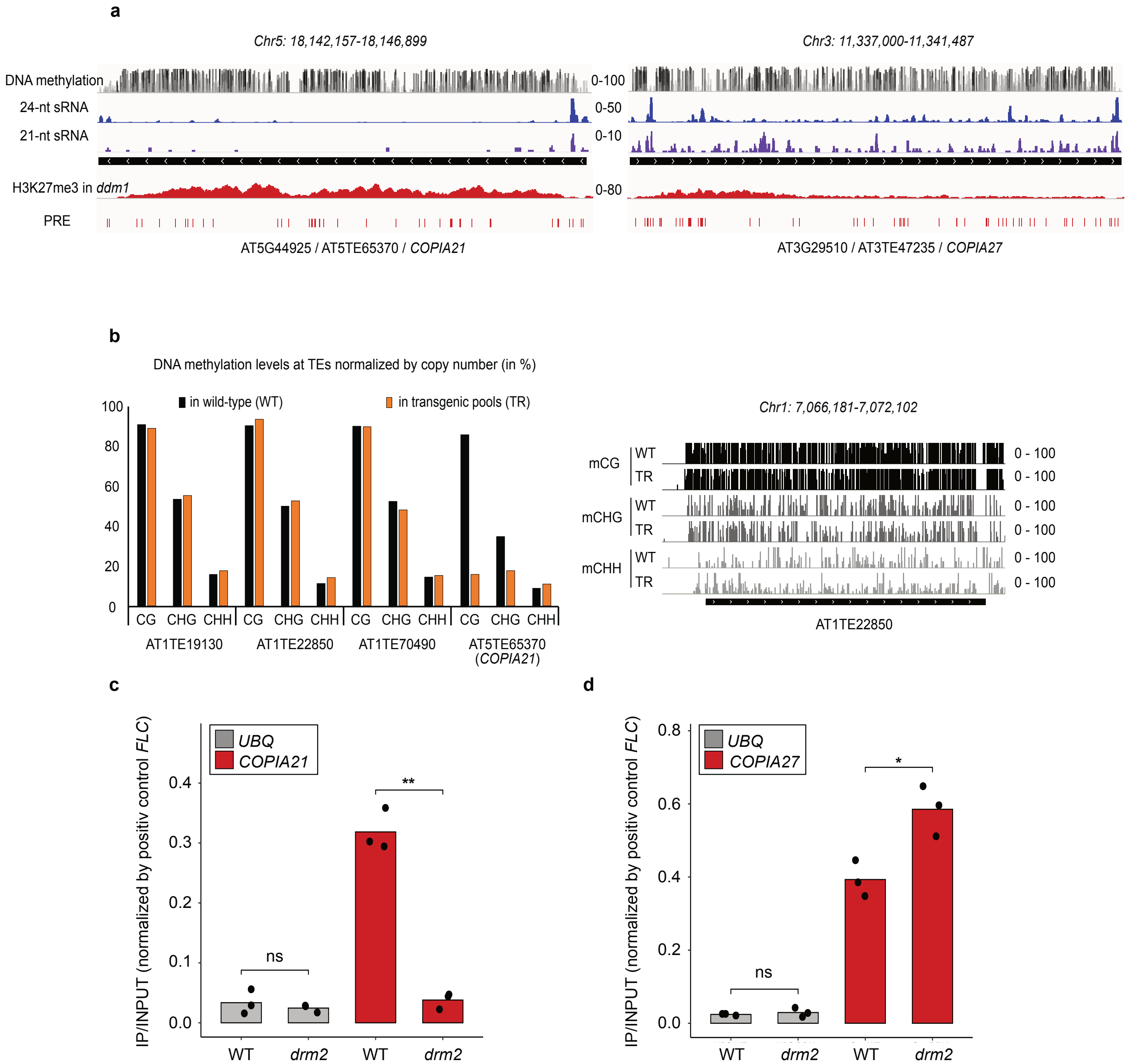

Supplementary Figure 1. Role of DRM2 in the establishment of *de novo* H3K27me3 at a neo-inserted TE transgenic sequence.
(a) Representative genome browser view of DNA methylation (CG: black, CHG: dark grey, CHH: light grey), 24-nt small RNAs levels (blue) and 21/22-nt small RNAs levels (purple) in wild-type, H3K27me3 levels in ddm1 mutant and Polycomb-Responsive Elements (PREs) (red) at *COPIA21* (**left panel**) and *COPIA27* (**right panel**). **(b) Left panel**: Bar plot showing the DNA methylation levels in each cytosine context at different endogenous TEs in WT (black bars) and in transgenic pools (orange bars). **Right panel**: Representative genome browser view of DNA methylation status (CG: black, CHG: dark grey, CHH: light grey) at *AT1TE22850* in wild-type (WT) and in transgenic pools (TR). **(c)** Bar plot showing H3K27me3 levels assessed by ChIP-qPCR at *COPIA21* in wild-type and *drm2 COPIA21* transgenic lines. IPs were normalized by the input and then normalized by the positive control *FLC*. Three independent biological replicates are shown. **(d)** Bar plot showing H3K27me3 levels assessed by ChIP-qPCR at *COPIA27* in wild-type and *drm2 COPIA27* transgenic lines. IPs were normalized by the input and then normalized by the positive control *FLC*. Three independent biological replicates are shown.

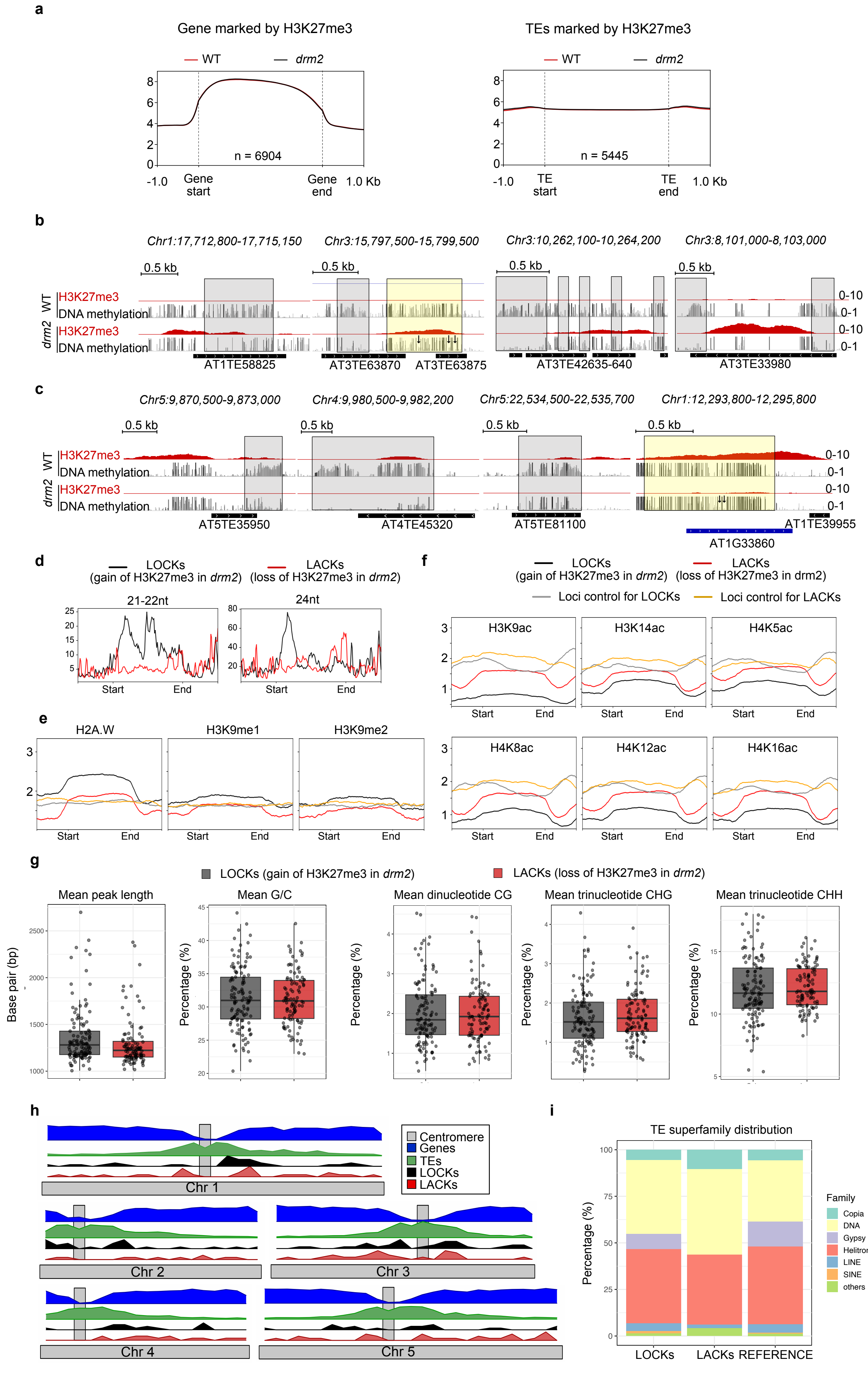

Supplementary Figure 2. Role of DRM2 in the recruitment of H3K27me3 at endogenous loci.
(See below for legend)

Supplementary Figure 2. Role of DRM2 in the recruitment of H3K27me3 at endogenous loci.
(a) Metagenes showing H3K27me3 level for all genes (**left panel**) and all TEs (**right panel**) marked by H3K27me3, in WT and *drm2* mutant. **(b)** Representative genome browser views showing H3K27me3 ChIP-seq and DNA methylation BS-seq data in WT and *drm2* mutant. Gain of H3K27me3 in *drm2* compared to WT is either associated with previously published DMRs (grey boxes) (*Stroud et al, 2013*) or DNA methylated regions that lose several methylated cytosines (yellow box; the arrows indicate the loss of several mCHH). **(c)** Representative genome browser views showing H3K27me3 ChIP-seq and DNA methylation BS-seq data in WT and *drm2* mutant. Loss of H3K27me3 in *drm2* compared to WT is either associated with previously published DMRs (grey boxes) (*Stroud et al, 2013*) or DNA methylated regions that lose several methylated cytosines (yellow box; the arrows indicate the loss of several mCHH). **(d)** Metaplots showing 21/22-nt and 24-nt small RNAs profiles at LOCKs (loci that gain H3K27me3 in *drm2*, black) and at LACKs (loci that loose H3K27me3 in *drm2*, red). **(e)** Metaplots showing H2A.W variant, H3K9me1 and H3K9me2 profiles at LOCKs (black), at LACKs (red) and at associated control regions (random loci of the same length in the genome). **(f)** Metaplots showing H3K9acetylation, H3K14ac, H4K5ac, H4K8ac, H4K12ac and H4K16ac profiles at LOCKs (black), at LACKs (red) and at associated control regions (random loci of the same size in the genome). **(g)** Box plots showing the mean of peak length, the mean of C and G nucleotides, the mean of CG dinucleotides, the mean of CHG trinucleotides and the mean of CHH trinucleotides at LOCKs (black) and LACKs (red). **(h)** Genomic distribution of the LOCKs (black), the LACKs (red), the genes (blue), the TEs (green) and the centromeric region (grey). **(i)** Stacked bar plot showing the distribution of TE superfamilies in LOCKs (black), LACKs (red), and in the whole genome as 'REFERENCE'.

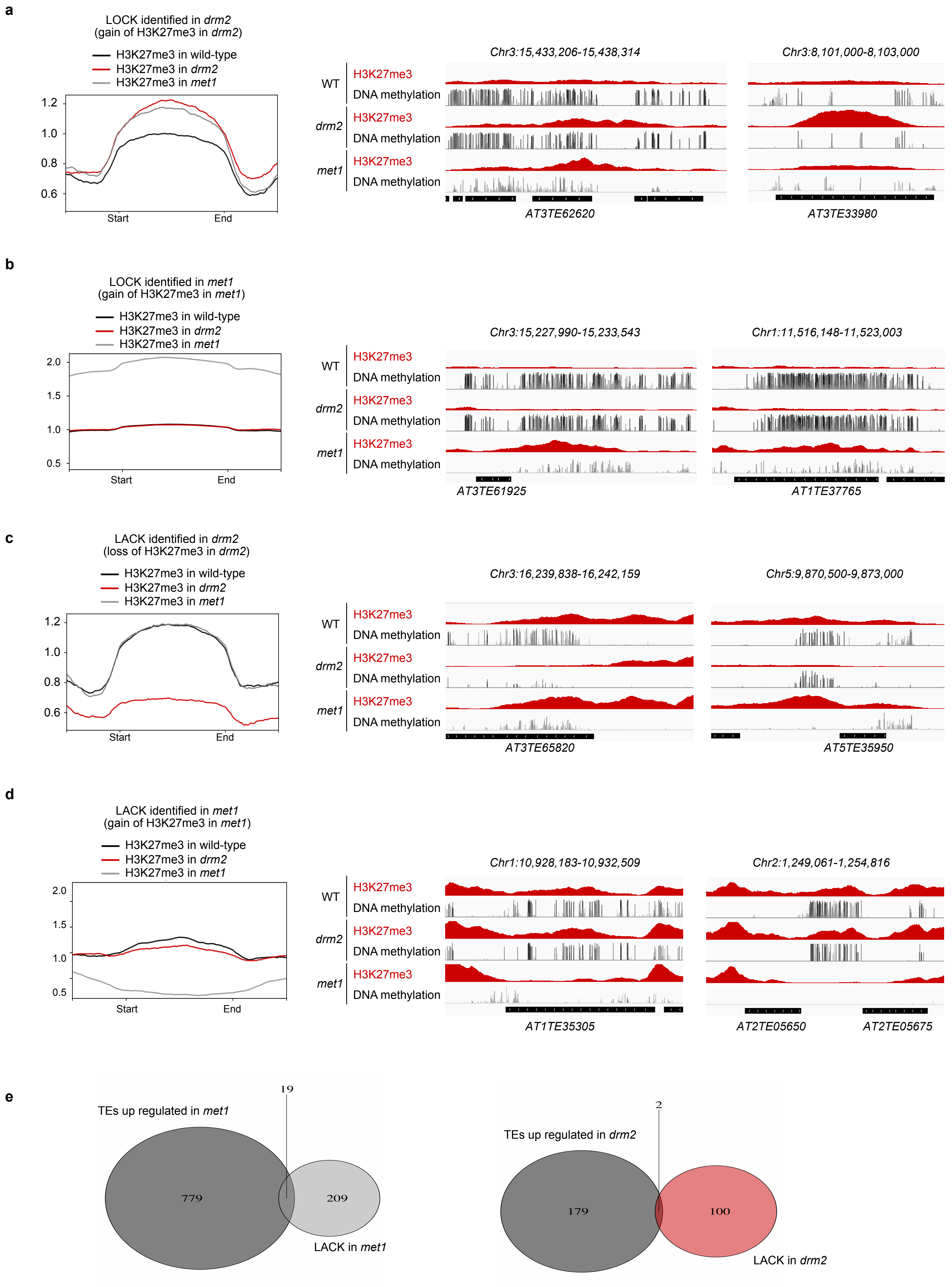

Supplementary Figure 3. Role of MET1 in the recruitment of H3K27me3 at endogenous loci.
(See below for legend)

Supplementary Figure 3. Role of MET1 in the recruitment of H3K27me3 at endogenous loci. loci.

(a) Metaplots showing H3K27me3 profiles in wild-type (black), *drm2* (red), and *met1* (grey) at LOCKs identified in *drm2* (loci that gain H3K27me3 in *drm2*). Representative genome browser views of LOCKs identified in *drm2*, showing H3K27me3 ChIP-seq and DNA methylation BS-seq data in WT, *drm2* and *met1* mutant. **(b)** Metaplots showing H3K27me3 profiles in wild-type (black), *drm2* (red), and *met1* (grey) at LOCKs identified in *met1* (loci that gain H3K27me3 in *met1*). Representative genome browser views of LOCKs identified in *met1*, showing H3K27me3 ChIP-seq and DNA methylation BS-seq data in WT, *drm2* and *met1* mutant. **(c)** Metaplots showing H3K27me3 profiles in wild-type (black), *drm2* (red), and *met1* (grey) at LACKs identified in *drm2* (loci that loose H3K27me3 in *drm2*). Representative genome browser views of LACKs identified in *drm2*, showing H3K27me3 ChIP-seq and DNA methylation BS-seq data in WT, *drm2* and *met1* mutant. **(d)** Metaplots showing H3K27me3 profiles in wild-type (black), *drm2* (red), and *met1* (grey) at LACKs identified in *met1* (loci that loose H3K27me3 in *met1*). Representative genome browser views of LACKs identified in *met1*, showing H3K27me3 ChIP-seq and DNA methylation BS-seq data in WT, *drm2* and *met1* mutant. **(e)** Left panel: Venn diagram showing intersection between up-regulated TEs in *met1* (*Zhao et al, 2022*) and our LACKs identified in *met1*. Right panel: Venn diagram showing intersection between up-regulated TEs in *drm2* (*Stroud et al, 2014*) and our LACKs identified in *drm2*.

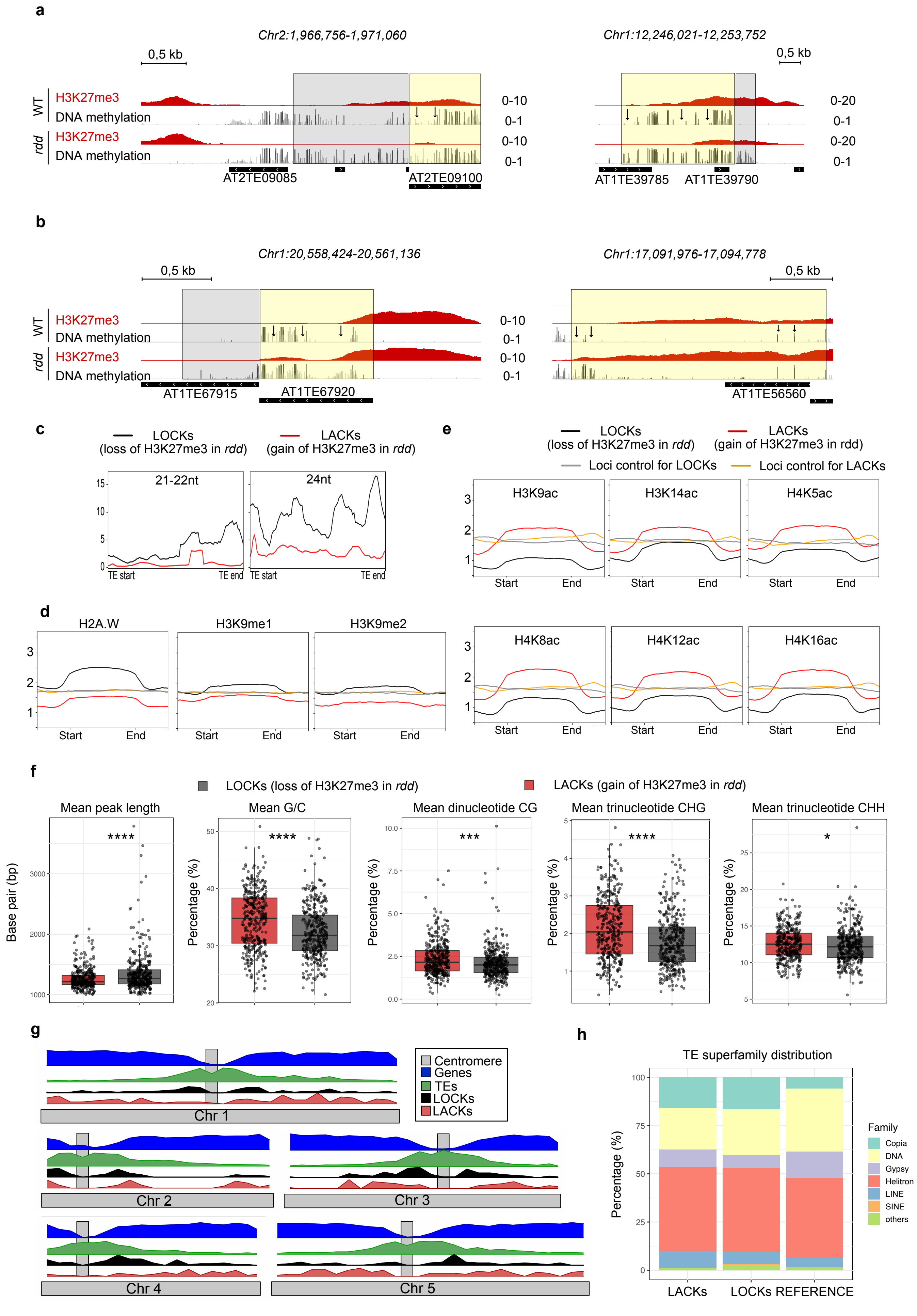

Supplementary Figure 4. DNA demethylation impacts H3K27me3 patterning at endogenous loci.
(See below for legend)

Supplementary Figure 4. DNA demethylation impacts H3K27me3 patterning at endogenous loci.
(a) Representative genome browser views showing H3K27me3 ChIP-seq and DNA methylation BS-seq data in WT and *rdd* mutant. Loss of H3K27me3 in *rdd* compared to WT is either associated with previously published DMRs (grey boxes) (*Duan et al, 2015*) or DNA methylated regions that gain several methylated cytosines (yellow box; the arrows indicate the gain of several mCHH). **(b)** Representative genome browser views showing H3K27me3 ChIP-seq and DNA methylation BS-seq data in WT and *rdd* mutant. Gain of H3K27me3 in *rdd* compared to WT is either associated with previously published DMRs (grey boxes) (*Duan et al, 2015*) or DNA methylated regions that gain several methylated cytosines (yellow box; the arrows indicate the gain of several mCHH). **(c)** Metaplots showing 21/22-nt and 24-nt small RNAs profiles at LOCKs (loci that lose H3K27me3 in *rdd*, black) and at LACKs (loci that gain H3K27me3 in *rdd*, red). **(d)** Metaplots showing H2A.W variant, H3K9me1 and H3K9me2 profiles at LOCKs (black), at LACKs (red) and at associated control regions (r random loci of the same length in the genome). **(e)** Metaplots showing H3K9acetylation, H3K14ac, H4K5ac, H4K8ac, H4K12ac and H4K16ac profiles at LOCKs (black), at LACKs (red) and at associated control regions (random loci of the same size in the genome). **(f)** Box plots showing the mean of peak length, the mean of C and G nucleotides, the mean of CG dinucleotides, the mean of CHG trinucleotides and the mean of CHH trinucleotides at LOCKs (black) and LACKs (red). **(g)** Genomic distribution of the LOCKs (black), the LACKs (red), the genes (blue), the TEs (green) and the centromeric region (grey). **(h)** Stacked bar plot showing distribution of TE superfamilies in LOCKs (black), LACKs (red), and in the whole genome as ‘REFERENCE’.

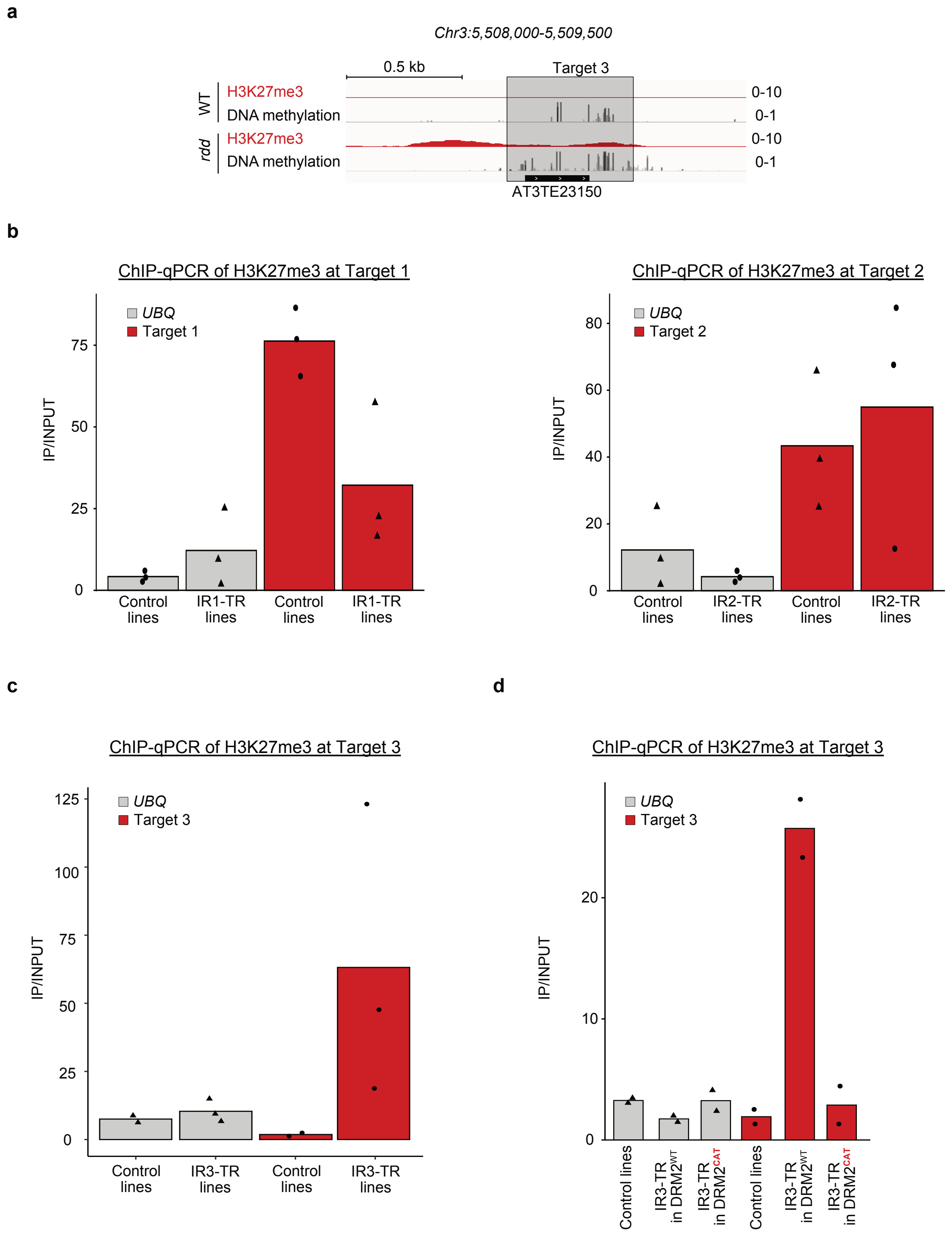

Supplementary Figure 5. Targeted DNA methylation by Inverted-Repeats (IR) reveals a dual role of DNA methylation on H3K27me3 patterning at endogenous TEs.
(a) Representative genome browser view showing H3K27me3 and DNA methylation in WT and *rdd* mutant. The region highlighted in grey corresponds to a DMR that was cloned as an inverted-repeat (IR) for the experiments shown in panels c and d. **(b)** ChIP-qPCR analysis of H3K27me3 levels in control and IR-transgenic lines at Target 1 (IR1, **left panel**) and Target 2 (IR2, **right panel**). This panel shows a biological replicate of the experiment presented in Fig.4 (ChIP replicate on three lines). Bar plots show the mean H3K27me3 levels from three independent control and transgenic lines. Each dot represents one transgenic line. Biological replicates are shown separately in Fig.4 and Fig.S4 due to differences in ChIP efficiencies between the two experimental batches. **(c)** ChIP-qPCR analysis of H3K27me3 levels in control and IR-transgenic lines at Target 3 (IR3). Bar plots show the mean H3K27me3 levels from three independent IR3 lines with each dot representing one line. **(d)** ChIP-qPCR analysis of H3K27me3 levels in $DRM2^{WT}$ or $DRM2^{CAT}$ T1 plants transformed with IR-control or IR3 constructs (pools of T1). Data were normalized to the positive control *FLC*; *UBQ* serves as a negative control.

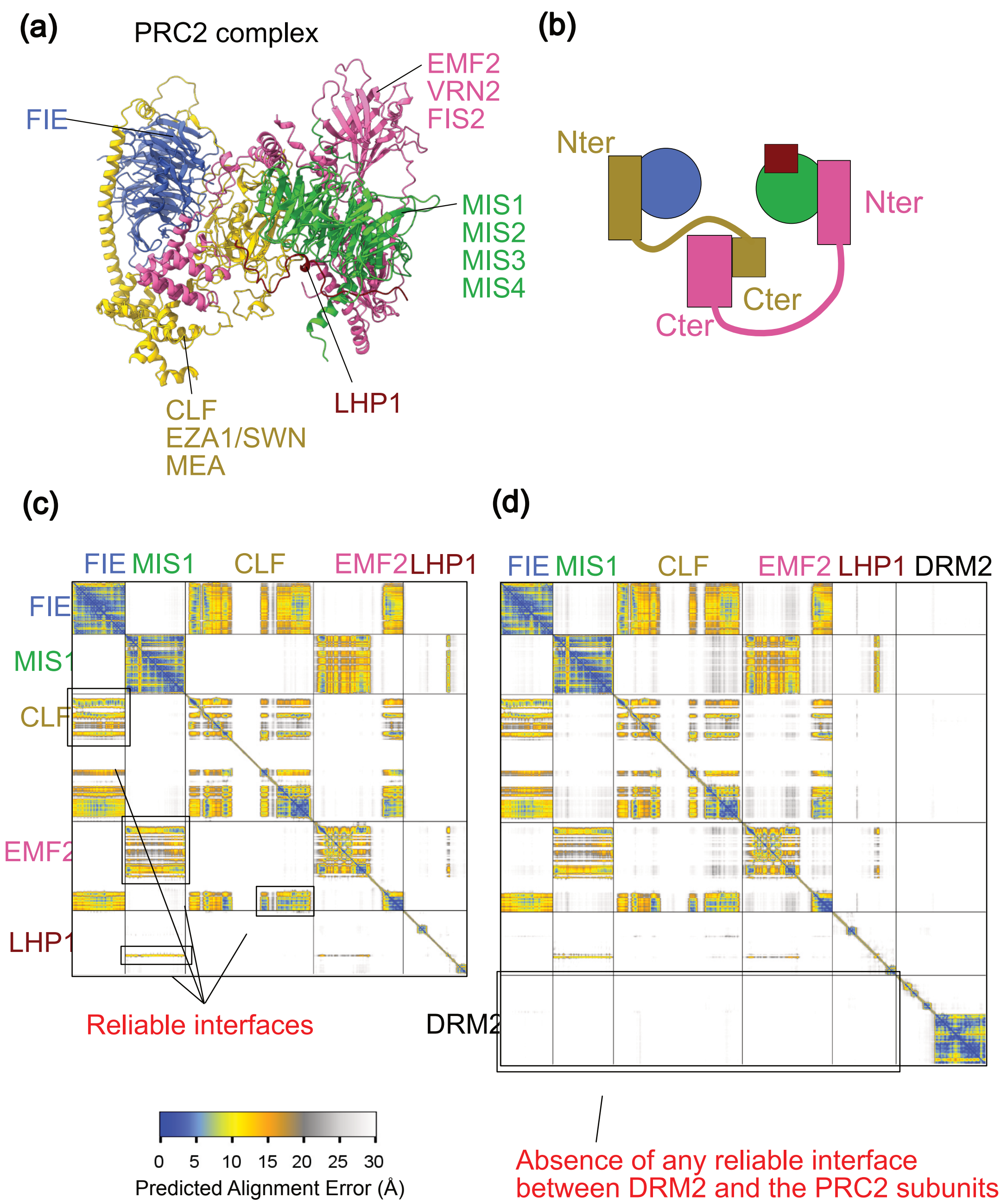

Supplementary Figure 6. AlphaFold3 predictions reveal no reliable interfaces between DRM2 and PRC2 subunits.
(**a**) Structural model of the Arabidopsis PRC2 complex, highlighting the core subunits FIE (blue), CLF (yellow) (can also be substituted by EZA1/SWN or MEA) , the MSI1 subunit (green) (can also be substituted by MSI2, MSI3 or MSI4), EMF2 (magenta) (can also be substituted by VRN2 or FIS2) and LHP1 (red). (**b**) Schematic representation of the N-terminal (Nter) and C-terminal (Cter) regions of PRC2 components, illustrating the relative positions of the interfaces and flexible regions. (**c**) Predicted Alignment Error (PAE) heatmap of the PRC2 subunits alone, showing multiple high-confidence interaction interfaces (blue/orange blocks). Arrows indicate well-supported interfaces recovered by AlphaFold3. (**d**) PAE heatmap of PRC2 subunits modelled together with DRM2, showing that DRM2 does not form any reliable interface with any PRC2 subunit (large white/grey region at the bottom right). Color scale: lower PAE values (blue) indicate high-confidence residue-residue distances, whereas higher PAE values (yellow/grey) reflect low-confidence or flexible regions.

Supplementary Table 1.

Primer alias	Primer sequence	Associated figure
COPIA27 Forward 1	ATGAACAGCGCATTGAGACC	Fig.1
COPIA27 Reverse 1	CATAGTGGTTTCCTTGGCCG	Fig.1
COPIA27 Forward 2	TACGGTCGAGGATCCACTTG	Fig.1
COPIA27 Reverse 2	CACCACACAGCCTCAGGATA	Fig.1
COPIA27 Forward 3	GCCGAGGACAAGGACATACT	Fig.1
COPIA27 Reverse 3	CTCCTCTTTGAGCTGGGTGA.	Fig.1
COPIA21 Forward	CGTGAGAGATGTGGTGAGGT	Supplementary Fig.1 and Fig.4
COPIA21 Reverse	ACAAAATCCACCGCTTCCAC	Supplementary Fig.1 and Fig.4
Target 1 Forward	TCACCATAGTCACCATCGGA	Fig.4
Target 1 Reverse	GCCAGAAGAGAAACAAGTAGCT	Fig.4
Target 2 Forward	TGTAAGAAGATATGAAGCCGCA	Fig.4
Target 2 Reverse	AATAGTTGTTTTGTGGCTCTTCC	Fig.4
Target 3 Forward	CACCATCCAATGTGTGTATGCGCC	Supplementary Fig.4
Target 3 Reverse	GGAACTAGGACAAAACTGGGAG	Supplementary Fig.4
UBQ Forward	TGAAGTCGTGAGACAGCGTTG	Fig.1, Fig.4, Supplementary Fig.1 and Supplementary Fig.4
UBQ Reverse	GGGCTTTCTCATTGTTGGTC	Fig.1, Fig.4, Supplementary Fig.1 and Supplementary Fig.4
FLC Forward	TTGCTTGATTAATTTGGGGTTT	Fig.1, Fig.4, Supplementary Fig.1 and Supplementary Fig.4
FLC Reverse	GCCGGTCTTCCATTTTGTTA	Fig.1, Fig.4, Supplementary Fig.1 and Supplementary Fig.4
