# Supplementary material for: DNA methylation either antagonizes or promotes Polycomb recruitment at transposable elements in Arabidopsis
Source: Nat Commun. 2026 Sep 2;17:10439. doi: 10.1038/s41467-026-77108-9 (PMC13631316; doi:10.1038/s41467-026-77108-9)
Supplement: Supplementary file 2 [file 41467_2026_77108_MOESM2_ESM.pdf]

nature portfolio

Peer Review File

DNA methylation either antagonizes or promotes Polycomb recruitment at transposable elements

Corresponding Author: Dr Angélique DELERIS

This file contains all reviewer reports in order by version, followed by all author rebuttals in order by version.

Version 0:

Reviewer comments:

Reviewer #1

(Remarks to the Author)
This research reported the potential interplay between DNA methylation and H3K27me3. Traditionally, DNA methylation and H3K27me3 are considered as two separated epigenetics marks located in TEs and genes, respectively. However, it is emerged that these two marks have crosstalk. In this research, the authors used Arabidopsis as a model system to investigate their crosstalk. The authors found that both the RdDM mutation drm2 or the DNA demethylase mutation rdd have effects on the H3K27me3 deposition. Interestingly, at some loci, the DNA methylation antagonizes H3K27me3 deposition, while at some loci, DNA methylation promotes H3K27me3. Overall, the story is very interesting and provide important insights into the evolutionary relationship between DNA methylation and H3K27me3. I have the following suggestions.
1. Is it possible to analysis the features of DNA methylation-dependent H3K27me3 loci and DNA methylation-repelled H3K27me3? An in-depth analysis is strongly suggested.
2. The current research only focused on the dependence of H3K27me3 by DNA methylation. Is is possible to have a reverse analysis? Does the DNA methylation status change in those two groups of TEs in H3K27me3 deficient mutant? For example, an analysis of DNA methylation in clf may provides some insights.

Reviewer #2

(Remarks to the Author)
Hure et al. claim to uncover a novel interplay between DNA methylation and H3K27me3 in the silencing of TEs, yet their findings raise several questions that require further clarification. While they report that DNA methylation can both promote and antagonize H3K27me3 deposition at different loci, their mechanistic insights remain incomplete.
1. The study focuses heavily on the drm2 mutant, but it is essential to determine whether alterations in H3K27me3 levels are observed in other DNA methylation mutants. Without this, it remains unclear whether PRC2 recruitment and catalysis are strictly dependent on DRM2.
2. The assertion that DNA methylation facilitates H3K27me3 deposition would be significantly strengthened by direct biochemical evidence of physical interactions between PRC2 and DRM2. Have the authors attempted co-immunoprecipitation or other relevant assays to support this claim?
3. The classification of TEs into “type 1-targets” and “type 2-targets” lacks depth. The authors should conduct a thorough analysis of these targets, considering TE superfamilies, chromatin state, insertion time, and sequence length to identify meaningful trends. Without this, the biological significance of their classification remains ambiguous.
4. The relationship between the genes in Fig. 2A and the so-called “type 1-targets” (line 200) is unclear, as is the connection between Fig. 2C and “type 2-targets” (line 205). These correlations must be explicitly addressed and quantified.
5. While the targeted DNA methylation approach is a step in the right direction, it falls short of providing definitive mechanistic insight. A more direct CRISPR-based DNA methylation editing system—such as DRM2 or MQ1—should be employed. This would allow the authors to determine whether changes in H3K27me3 levels are truly dependent on DRM2. If they are, MQ1-mediated methylation should increase DNA methylation without affecting H3K27me3 levels. Additionally, testing how different sequence contexts influence H3K27me3 deposition is critical for strengthening their conclusions.
Overall, while the study presents intriguing observations, its mechanistic framework remains insufficient. Without addressing these fundamental gaps, the impact of the work remains limited.

Reviewer #3

(Remarks to the Author)
DNA methylation and Polycomb are associated with transcriptional repression through modification of either DNA or Histone H3 containing chromatin. Originally described as independent and mutually antagonistic systems (depending on CG density in vertebrates), it has now become very clear that they impact on each other's genomic landscape by altering the local deposition and higher order chromatin structures associated with these modifications. This includes the concept of modification spreading, where loss of DNA methylation can cause a reprogramming of the Polycomb landscape as evidenced the appearance of H3K27me3 in formally methylated regions either locally or globally, including repeat regions. At the same time loss of Polycomb mediated H3K27me3 in model systems can result in the de novo methylation of CG rich regions, which can have functional outcomes. In certain situations, the absence of one repression system can be partially compensated by the other.

Here, Hure and colleagues report on the phenomena in plants, with a focus on the silencing of transposable elements (TE's). Using the model plant Arabidopsis, which exhibits DNA methylation in three contexts; CG, CHG, and CHH (H = A, T, C), they investigated the roles of DNA methylation (CHH) and H3K27me3 deposition in silencing Copia TEs. In plants, domains rearranged methyltransferase 2 (DRM2) preferentially mediates CHH methylation, a substrate specificity distinct from that of mammalian DNA methyltransferases. They demonstrate that a specific transgenic TE can recruit both H3K27me3 and CHH DNA methylation de novo, and H3K27me3 deposition can be dependent on the DRM2 enzyme. They suggest this may be due to a positive crosstalk between the two systems in the context of TE neo-insertion. They also show for other TE's that DNA methylation is antagonistic to H3K27me3 accumulation.

So, what is not clear is the context in which Copia elements exhibit Drm2 dependent recruitment of H3K27me3 or gain H3K27me3 upon loss of Drm2? What differentiates these two classes of elements? This is difficult to address as the Arabidopsis genome contains multiple Copia copies, they need to demonstrate if recruitment is locus specific or dependent on the sequences of various Copia elements and if potential corresponding siRNAs have a role? The Copia element they used (Figure 2) as a transgene exhibits both DNA methylation (CHH) and H3K27me3 deposition in WT plants, can they use a variant in the transgene assay that gains H3K27me3 in the Drm2 mutant as a transgene? Then directly compare the properties of the two by analysing recombinant versions. This should address more precisely the determinants of acquisition and loss of these modifications.

Version 1:

Reviewer comments:

Reviewer #1

(Remarks to the Author)
My concerns have been addressed. I am satisfied with this revision and support publication of this version.

Reviewer #3

(Remarks to the Author)
The authors have addressed my comments very well, bravo. However, by shoe-horning all the new data into the original manuscript, it becomes hard to digest. The summary diagram helps, Figuure 5. But I suggest it should addressed/explained more directly in the discussion for the benefit of the reader, rather than in passing in the discussion. More clarity is needed.

Open Access This Peer Review File is licensed under a Creative Commons Attribution 4.0 International License, which permits use, sharing, adaptation, distribution and reproduction in any medium or format, as long as you give appropriate credit to the original author(s) and the source, provide a link to the Creative Commons license, and indicate if changes were made.
In cases where reviewers are anonymous, credit should be given to 'Anonymous Referee' and the source.
The images or other third party material in this Peer Review File are included in the article's Creative Commons license, unless indicated otherwise in a credit line to the material. If material is not included in the article's Creative Commons license and your intended use is not permitted by statutory regulation or exceeds the permitted use, you will need to obtain permission directly from the copyright holder.
To view a copy of this license, visit https://creativecommons.org/licenses/by/4.0/

RESPONSE TO REVIEWERS' COMMENTS

Summary of the point-by-point response for the Reviewers

We thank the reviewers for their constructive evaluations. We believe we have comprehensively addressed the essential points that have been raised.

First, as suggested by Reviewers #1, #2 and #3, we now provide an extensive genome-wide analysis identifying the genetic and epigenetic features that distinguish the two classes of loci at which DNA methylation either promotes H3K27me3 (now referred to as LACK for *Loci of Association between Cytosine methylation and K27me3*) or antagonizes H3K27me3 (now referred to these as LOCK for *Loci of Opposition between Cytosine methylation and K27me3*). These analyses include chromatin marks, histone variants, sequence composition and length, TE family distribution, genomic distribution, and siRNA profiles. They are further supported by experimental testing of an additional COPIA element in the neo-insertion assay, as suggested by Reviewer #3.

Second, as suggested by Reviewer#2, we have directly tested the mechanistic basis of the relationship between DNA methylation and PRC2 recruitment: through the use of a catalytically inactive DRM2 mutant, we demonstrated that DNA methylation itself—rather than the DRM2 protein—is required for H3K27me3 deposition. This is further supported by analyses in another DNA methyltransferase mutant also suggested by Reviewer#2.

Together, these additions substantially strengthen the conceptual and mechanistic conclusions of the manuscript.

REVIEWER COMMENTS (comments are in blue and our responses are in black; revised sections are highlighted in grey in the manuscript)

Reviewer #1 (Remarks to the Author):

This research reported the potential interplay between DNA methylation and H3K27me3.

Traditionally, DNA methylation and H3K27me3 are considered as two separated epigenetics marks located in TEs and genes, respectively. However, it is emerged that these two marks have crosstalk. In this research, the authors used Arabidopsis as a model system to investigate their crosstalk. The authors found that both the RdDM mutation drm2 or the DNA demethylase mutation rdd have effects on the H3K27me3 deposition. Interestingly, at some loci, the DNA methylation antagonizes H3K27me3 deposition, while at some loci, DNA methylation promotes H3K27me3. Overall, the story is very interesting and provide important insights into the evolutionary relationship between DNA methylation and H3K27me3.
I have the following suggestions.

We thank the reviewer for evaluating the manuscript and for constructive suggestions, which we have addressed to the best of our ability.

1. Is it possible to analysis the features of DNA methylation-dependent H3K27me3 loci and DNA methylation-repelled H3K27me3? An in-depth analysis is strongly suggested.

We now have performed the requested in-depth analysis of the features of the DNA methylation-dependent H3K27me3 loci (which we now refer to as "LACK" for **L**oci of **A**ssociation between **C**ytosine methylation and **K**27me3) and DNA methylation-repelled H3K27me3 (which we now refer to "LOCK" **L**oci of **O**pposition between **C**ytosine methylation and **K**27me3): we have analyzed different chromatin marks and histone variants (21/22-nt and 24-nt small RNA accumulation, H3K9me2, H3K9me1, H2AW, H2Aub, H2AZ, H3ac, H4ac, in addition to DNA methylation in all three sequence contexts which was presented before), sequence composition (C, CG, CHG, CHH content, Polycomb-responsive element "PRE" content) and length, TE superfamily distribution and genomic distribution.

We have found common features specific of the LOCK (whether they are defined in *drm2* or *rdd*) and common features of the LACKS (whether they are defined in *drm2* or *rdd*) respectively and significant differences between LOCK and LACK. Essentially, LACKS tend to be less DNA methylated than LOCKS with less small RNA accumulation, less constitutive heterochromatin marks but more facultative heterochromatin marks/hallmarks of stress-responsive genes such as H2Aub, H2AZ, H3/H4ac; they tend to be shorter (consistent with short TEs being less DNA methylated) and with less PREs motifs than LOCKs.

<u>The analyses are presented in Figures 2e-h, Supplementary Figures 2d-i as well as Figures 3e-h and Supplementary Figures 4c-h.</u>

They are described in the revised manuscript as below as indicated below and discussed in the appropriate section.

- <u>Between line 205 and line 218</u> (meta-analysis of LOCKs and LACKs defined with WT versus *drm2* comparison= DRM2-LOCKs and DRM2-LACKs)

"*Additional meta analyses using published datasets…*
… Together, this points to particular epigenetic and genetic signatures of LACK versus LOCK which likely both contribute to explain the locus-specific and opposite effects of DRM2 on H3K27me3 deposition".

- <u>Between line 258 and line 279</u> (meta-analysis of LOCK and LACK defined with WT versus *rdd* comparison)

"To further characterize these subsets, we performed meta-analyses …

… The opposing outcomes at these loci are associated with distinct genetic and epigenetic signatures shared across the corresponding DRM2- and RDD-defined classes, and point to locus-specific rules that govern the interplay between DNA methylation dynamics and Polycomb targeting."

- <u>Discussion between line 347 and line 372</u>

"Taken together, our results point to locus-specific rules for H3K27me3 deposition, which would either require DNA methylation or be antagonized by its presence (Fig. 5b). We have named these two classes…

… For instance, it could be predicted that LOCK-specific TF which recognize PREs are sensitive to DNA methylation while LACK-specific TF, associated to different sequence motifs bind to DNA methylation (DRM2-dependent or not based on the nature of the sequence motif).

2. The current research only focused on the dependence of H3K27me3 by DNA methylation. Is is possible to have a reverse analysis? Does the DNA methylation status change in those two groups of TEs in H3K27me3 deficient mutant? For example, an analysis of DNA methylation in clf may provides some insights.

We thank the reviewer for raising this insightful point. We performed the suggested analysis by examining DNA methylation levels at LOCK and LACK elements in the *clf* mutant—which is impaired in H3K27me3 deposition—using a previously published dataset from our group (Rougée et al., 2021). We did not detect any difference in DNA methylation levels between wild type and *clf* as shown in the Figure below.

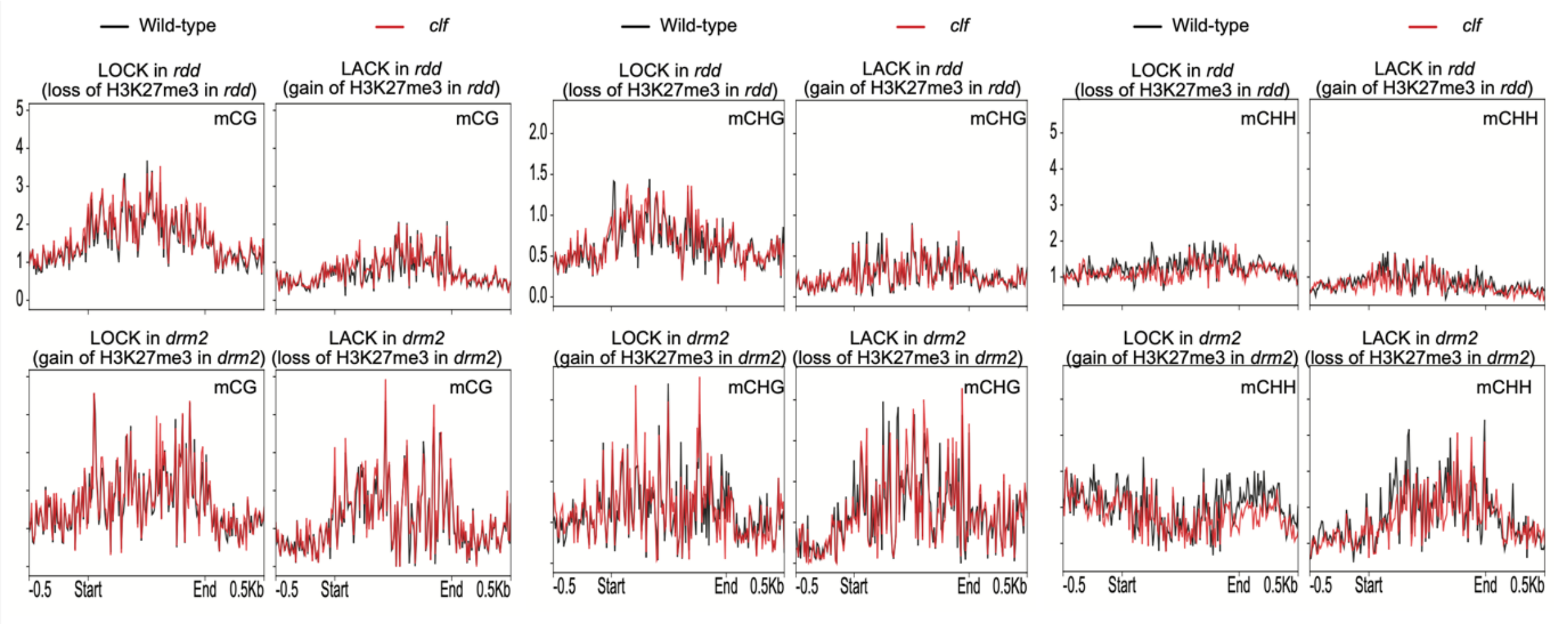

Figure. DNA methylation is not impaired in *clf* mutant at LACKs and LOCKs identified in *rdd* and *drm2*. Metagene showing DNA methylation levels in each context (mCG at left, mCHG at middle and mCHH at right) in wild-type (black) and in *clf* mutant (red), at previously identified regions. LOCKs and LACKs identified in *rdd* are at the top, respectively left and right while LOCKs and LACKs identified in *drm2* are at the bottom, left and right respectively.

However, we consider that this result is not substantial enough to be presented as a standalone figure, and incorporating it into the main text would not meaningfully advance our manuscript, which is specifically focused on the impact of DNA methylation on H3K27me3 deposition. Furthermore, a comprehensive and rigorous investigation of this question would require dedicated experimental strategies, such as targeted H3K27me3 epigenome editing, to circumvent the pleiotropic developmental defects associated with currently available PRC2 mutants (e.g., embryonic arrest in *mea*, undifferentiated callus in *swn-clf*). Such work would constitute a separate and substantial study, particularly given that the specific H3K27 methyltransferases acting at TEs have not yet been clearly identified.

Reviewer #2 (Remarks to the Author):

Hure et al. claim to uncover a novel interplay between DNA methylation and H3K27me3 in the silencing of TEs, yet their findings raise several questions that require further clarification. While they report that DNA methylation can both promote and antagonize H3K27me3 deposition at different loci, their mechanistic insights remain incomplete.

We thank the reviewer for evaluating our manuscript and for constructive suggestions, which we have addressed to the best of our ability.

1. The study focuses heavily on the drm2 mutant, but it is essential to determine whether alterations in H3K27me3 levels are observed in other DNA methylation mutants. Without this, it remains unclear whether PRC2 recruitment and catalysis are strictly dependent on DRM2.

We agree with the reviewer that assessing additional DNA methylation mutants is essential to determine whether the observed alterations in H3K27me3 are specific to *drm2* or reflect a broader dependency on DNA methylation.

- We first addressed this point by analyzing a *drm2* catalytically inactive mutant in which the DRM2 protein accumulates and localizes similarly to wild type. This analysis showed that the effects on H3K27me3 recruitment require DRM2-mediated DNA methylation rather than the presence of the DRM2 protein itself (see the Response to comment #5 for more details).

- To further test whether PRC2 recruitment depends exclusively on DRM2-mediated methylation or can also be influenced by other methyltransferases, we profiled H3K27me3 in *met1* mutants. This experiment revealed two classes of loci:

1) those at which H3K27me3 is repelled by MET1-dependent methylation ("LOCK" i.e. **L**oci of **O**pposition between **C**ytosine methylation and **K**27me3, as defined in *met1*)
2) those at which H3K27me3 is promoted by MET1-dependent methylation ("LACK" i.e. for **L**oci of **A**ssociation between **C**ytosine methylation and **K**27me3, as defined in *met1*).

Notably, the LACKs defined in *met1* (where H3K27me3 is promoted by MET1-mediated methylation) constitute a distinct set of loci from LACKs defined in *drm2 (i.e.* these at which H3K27me3 is promoted by DRM2-mediated methylation.
These new analyses are presented in an additional Supplementary Figure 3 related to Figure 2, with 5 panels.
Analyses have been incorporated into the revised manuscript, as an additional paragraph, between Line 219 and Line 235.
L.219: "*Finally, to determine whether PRC2 recruitment depends specifically on DRM2 or whether other DNA methyltransferases can serve similar roles,…*
…Together, these results show that PRC2 recruitment and activity can be influenced by DNA methylation mediated by DRM2 or by other methyltransferases, depending on the locus."L.235

2. The assertion that DNA methylation facilitates H3K27me3 deposition would be significantly strengthened by direct biochemical evidence of physical interactions between PRC2 and DRM2. Have the authors attempted co-immunoprecipitation or other relevant assays to support this claim?

We thank the reviewer for this valuable suggestion. Despite several attempts, we have not yet been able to identify suitable experimental conditions to successfully perform co-immunoprecipitation assays between PRC2 and DRM2. In the timeframe of the revisions (>6 months), we successfully generated F1 plants carrying simultaneously a GFP-tagged functional version of SWN and a Myc-tagged functional version of DRM2 as well as F1 plants carrying simultaneously a GFP-tagged functional version of CLF and a Myc-tagged functional version of DRM2 . While we could detect the fusion proteins in the parental lines by Western blot on total extracts, we could not detect them in the F1 either by western nor by immunoprecipitation. Possibly, we did not start with enough plant material or there was silencing of the proteins upon putting the transgenes together in the F1 (possible co-suppression). It would be necessary to repeat the crosses and wait for additional months to repeat this and with no guarantee of success. While we agree that such experiments would be highly informative, they would require substantial additional work to generate and validate the necessary plant material (including to tag and clone additional PRC2 sub-units, which we would love to do in future endeavors).

However, following the reviewer's comment to attempt other relevant assays, we initiated a new collaboration to explore potential structural interactions between PRC2 and DRM2 using AlphaFold-based predictions**.** We carried out these predictions using both full-length PRC2 subunits modeled within the complete complex using AlphaFold3 (Supplementary. Fig. 5), and, to increase sensitivity, by systematically evaluating pairwise interactions between shorter protein fragments following the methodology developed in (Bret et al Nat Comm 2024 doi: 10.1038/s41467-023-44288-7) (Supplementary Table 1). These analyses indicate that none of the PRC2 subunits, when assembled in the full PRC2 complex, are predicted to form stable physical interactions with DRM2. This observation supports the idea that any functional association between these machineries is likely indirect, potentially mediated by bridging proteins or by methyl-DNA–binding factors that remain to be identified. This would be in line with our results using a *drm2* catalytic mutant which demonstrate that it is DNA methylation, not DRM2 protein itself, that influences PRC2 recruitment.

Nevertheless, we also cannot rule out the possibility that a physical interaction might depend on post-translational modifications, since they are not reliably captured by current AlphaFold-based predictions. However, consistent with Alphafold predictions, there was no PRC2 sub-unit among the proteins associated with DRM2 identified by Mass Spectrometry (Zhong et al., 2014, DOI:10.1016/j.cell.2014.03.056)

The possibility that such interactions are locus-specific or occur within sub-stoichiometric complexes exists, which could make their detection by co-immunoprecipitation particularly difficult. Taken together, and considering the experimental setup and time required to develop additional genetic tools, we believe that an extensive biochemical investigation of this question would be beyond the scope of the present study but should be investigated in future endeavours.

Alphafold analyses are presented and detailed in Supplementary Fig.6 and Supplementary Table 1. The discussion as above was included in the discussion section between line 372 and line 383.

L.372: *This does not exclude physical interactions between PRC2 and DRM2 or the DNA methylation machineries in a locus-specific manner or in a context-specific manner (for example, during neo-insertion events). However, our results using a drm2 catalytic mutant demonstrate that it is DNA methylation, not DRM2 protein itself, that influences PRC2 recruitment. Moreover, Alphafold predictions indicate* that *the direct interactions between*

DRM2 and the various PRC2 subunits are unlikely (Supplementary Figure 6). Therefore, we propose that any potential physical association between these machineries is likely to be ***indirect****, mediated through* ***DNA methylation recognition*** *and involving* ***bridging factors*** *—such as transcription factors recognizing PREs or methyl-DNA–binding proteins—that remain to be identified (Fig. 5b). Given their role in PRC2 recruitment*[28] *, it is also possible that non-coding RNAs associated with DNA methylation could also be important in the positive crosstalk between DNA methylation and PRC2.* L.383

3. The classification of TEs into "type 1-targets" and "type 2-targets" lacks depth. The authors should conduct a thorough analysis of these targets, considering TE superfamilies, chromatin state, insertion time, and sequence length to identify meaningful trends. Without this, the biological significance of their classification remains ambiguous.

We now have performed the requested in-depth analysis of the features of the DNA methylation-dependent H3K27me3 loci (which we now refer to as "LACK" for **L**oci of **A**ssociation between **C**ytosine methylation and **K**27me3) and DNA methylation-repelled H3K27me3 (which we now refer to "LOCK" **L**oci of **O**pposition between **C**ytosine methylation and **K**27me3): we have analyzed different chromatin marks and histone variants (21/22-nt and 24-nt small RNA accumulation, H3K9me2, H3K9me1, H2AW, H2Aub, H2AZ, H3ac, H4ac, in addition to DNA methylation in all three sequence contexts which was presented before), sequence composition (C, CG, CHG, CHH content, Polycomb-responsive element "PRE"_content) and length, TE superfamily distribution and genomic distribution.

We have found common features specific of the LOCK (whether they are defined in *drm2* or *rdd*) and common features of the LACKS (whether they are defined in *drm2* or *rdd*) respectively and significant differences between LOCK and LACK. Essentially, LACKS tend to be less DNA methylated than LOCKS with less small RNA accumulation, less constitutive heterochromatin marks but more facultative heterochromatin marks/hallmarks of stress-responsive genes such as H2Aub, H2AZ, H3/H4ac; they tend to be shorter (suggesting they may represent older degenerated TEs and consistent with shorter TEs exhibiting reduced DNA methylation, Dombey et al., 2025) and with less PREs motifs than LOCKs.

<u>The analyses are presented in Figures 2e-h, Supplementary Figures 2d-i as well as Figures 3e-h and Supplementary Figures 4c-h.</u>

They are described in the revised manuscript as below as indicated below and discussed in the appropriate section.

- <u>Between line 205 and line 218</u> (meta-analysis of LOCKs and LACKs defined with WT versus *drm2* comparison= DRM2-LOCKs and DRM2-LACKs)

"Additional meta analyses using published datasets…

… Together, this points to particular epigenetic and genetic signatures of LACK versus LOCK which likely both contribute to explain the locus-specific and opposite effects of DRM2 on H3K27me3 deposition".

- <u>Between line 258 and line 279</u> (meta-analysis of LOCK and LACK defined with WT versus *rdd* comparison)

"To further characterize these subsets, we performed meta-analyses …

… The opposing outcomes at these loci are associated with distinct genetic and epigenetic signatures shared across the corresponding DRM2- and RDD-defined classes, and

point to locus-specific rules that govern the interplay between DNA methylation dynamics and Polycomb targeting."

- <u>Discussion between line 347 and line 372</u>

"Taken together, our results point to locus-specific rules for H3K27me3 deposition, which would either require DNA methylation or be antagonized by its presence (Fig. 5b). We have named these two classes…
… For instance, it could be predicted that LOCK-specific TF which recognize PREs are sensitive to DNA methylation while LACK-specific TF, associated to different sequence motifs bind to DNA methylation (DRM2-dependent or not based on the nature of the sequence motif).

4. The relationship between the genes in Fig. 2A and the so-called "type 1-targets" (line 200) is unclear, as is the connection between Fig. 2C and "type 2-targets" (line 205). These correlations must be explicitly addressed and quantified.

With the reviewer's comment, we realized that the initial version of the manuscript lacked clarity in how the different subsets were defined. We have now explicitly clarified the four subsets in the revised text.
-The DNA methylation-dependent H3K27me3 loci (DRM2-LACK) and DNA methylation-repelled H3K27me3 (DRM2-LOCK) as defined by WT versus *drm2* mutant comparisons
-The DNA methylation-dependent H3K27me3 loci (RDD-LACK) and DNA methylation-repelled H3K27me3 (RDD-LOCK) as defined by WT versus rdd mutant comparisons (previously referred to as Type 2 and Type 1 targets respectively)

For clarity, we added the following sentences <u>between Lines 268 and 273</u>:

L.268: *Importantly, RDD-LACKs and RDD-LOCKs are distinct from the corresponding DRM2-defined classes, consistent with their fundamentally different behaviors in wild type: DRM2-LACKs are H3K27me3-marked (or weakly marked) and lose H3K27me3 in drm2, whereas RDD-LACKs are H3K27me3-unmarked (or weakly marked) and gain H3K27me3 in rdd; conversely, DRM2-LOCKs are H3K27me3-unmarked and gain H3K27me3 in drm2, whereas RDD-LOCKs are H3K27me3-marked and lose it in rdd.* L.273

5. While the targeted DNA methylation approach is a step in the right direction, it falls short of providing definitive mechanistic insight. A more direct CRISPR-based DNA methylation editing system—such as DRM2 or MQ1—should be employed. This would allow the authors to determine whether changes in H3K27me3 levels are truly dependent on DRM2.
If they are, MQ1-mediated methylation should increase DNA methylation without affecting H3K27me3 levels.

We thank the reviewer for raising this insightful point. To address this question, we used an alternative but equally rigorous approach to the CRISPR-based systems suggested by the reviewer. Specifically, we took advantage of a previously published DRM2 catalytic mutant (Henderson, Deleris et al., 2010) that is impaired in methyltransferase activity but retains normal DRM2 protein accumulation and subcellular localization. This system allowed us to disentangle effects driven by DNA methylation from those potentially attributable to the DRM2 protein itself.

We aimed to transform this catalytic mutant with our inverted-repeat constructs (IR1 and IR2), which produce small RNAs and induce ectopic DNA methylation at defined loci. To further strengthen our conclusions, we also designed an additional hairpin targeting a third locus

corresponding to a LACK (as defined by WT vs. *rdd* comparisons) (Supplementary Figure 5a). As observed for our second target, induction of ectopic DNA methylation at this third site again resulted in a gain of H3K27me3 (Supplementary Figure 5c), reinforcing the conclusion that *de novo* DNA methylation is sufficient to promote H3K27me3 deposition.

Thus, we introduced IR1, IR2, IR3, as well as *COPIA21*, into both DRM2-WT/*drm2* plants (complementation line) and DRM2-Cat/*drm2* plants (catalytically inactive but protein-competent). Across all targets—Target 1 (a LOCK, showing loss of H3K27me3) and Targets 2 and 3 (both LACKs, showing gain of H3K27me3)—: only plants capable of depositing DNA methylation showed the corresponding H3K27me3 changes. In contrast, the DRM2 catalytic mutant, despite expressing and localizing DRM2 protein normally, failed to establish the methylation-dependent changes in H3K27me3.

Together, these results demonstrate that **it is DNA methylation itself, and not the presence of DRM2 protein, that drives PRC2 recruitment and H3K27me3 deposition.** These findings fully support, and are consistent with, the conclusions already presented in Response to Comment 1.

They are presented as new panels in Fig.4e and Supplementary Fig.5a,c,d and described <u>between lines 293 and 300</u> of the revised MS as well as in <u>between lines 314 and 320</u> and mentioned again in the discussion.

L.293 *"To uncouple the role of DNA methylation from the role of DRM2 protein per se…*
… Thus, it is DNA methylation, rather than DRM2 itself, that antagonizes H3K27me3 deposition"
…
"Importantly, in DRM2^{WT} lines, upon inverted-transgene introduction…
*…confirming that it is DNA methylation and not DRM2 itself that promotes H3K27me3 at LACK."*L.320

<u>Reviewer #3 (Remarks to the Author):</u>

DNA methylation and Polycomb are associated with transcriptional repression through modification of either DNA or Histone H3 containing chromatin. Originally described as independent and mutually antagonistic systems (depending on CG density in vertebrates), it has now become very clear that they impact on each other's genomic landscape by altering the local deposition and higher order chromatin structures associated with these modifications. This includes the concept of modification spreading, where loss of DNA methylation can cause a reprogramming of the Polycomb landscape as evidenced the appearance of H3K27me3 in formally methylated regions either locally or globally, including repeat regions. At the same time loss of Polycomb mediated H3K27me3 in model systems can result in the de novo methylation of CG rich regions, which can have functional outcomes. In certain situations, the absence of one repression system can be partially compensated by the other.

Here, Hure and colleagues report on the phenomena in plants, with a focus on the silencing of transposable elements (TE's). Using the model plant Arabidopsis, which exhibits DNA methylation in three contexts; CG, CHG, and CHH (H = A, T, C), they investigated the roles of DNA methylation (CHH) and H3K27me3 deposition in silencing Copia TEs. In plants,

domains rearranged methyltransferase 2 (DRM2) preferentially mediates CHH methylation, a substrate specificity distinct from that of mammalian DNA methyltransferases. They demonstrate that a specific transgenic TE can recruit both H3K27me3 and CHH DNA methylation de novo, and H3K27me3 deposition can be dependent on the DRM2 enzyme. They suggest this may be due to a positive crosstalk between the two systems in the context of TE neo-insertion. They also show for other TE's that DNA methylation is antagonistic to H3K27me3 accumulation.

So, what is not clear is the context in which C**opia** elements exhibit Drm2 dependent recruitment of H3K27me3 or gain H3K27me3 upon loss of Drm2? What differentiates these two classes of elements? This is difficult to address as the Arabidopsis genome contains multiple Copia copies, they need to demonstrate if recruitment is locus specific or dependent on the sequences of various Copia elements and if potential corresponding siRNAs have a role? The Copia element they used (Figure 2) as a transgene exhibits both DNA methylation (CHH) and H3K27me3 deposition in WT plants, can they use **a variant** in the transgene assay that gains H3K27me3 in the Drm2 mutant as a transgene? Then directly compare the properties of the two by analysing recombinant versions. This should address more precisely the determinants of acquisition and loss of these modifications.

We thank the reviewer for evaluating the manuscript and the constructive suggestions which we have addressed to the best of our ability.

We agree that is important to differentiate the two classes of elements that either exhibit DRM2-dependent recruitment of H3K27me3 (which we now refer to as LACK for Loci of Association of Cytosine methylation and H3K27me3) or Drm2 dependent gain of H3K27me3 (which we now refer to as "LOCK" for Loci of Opposition between Cytosine methylation and K27me3). We want to emphasize that this dual effect of DNA methylation on H3K27me3 deposition is not restricted to Copia elements but is observed across multiple TE superfamilies genome-wide. Hence, to investigate what differentiates the two classes, we performed a **comprehensive genome-wide analysis** (not limited to Copia TEs) by integrating of **a broad set of epigenetic features** (21/22-nt and 24-nt small RNA accumulation, diverse histone marks and histone variants: H3K9me2, H3K9me1, H2AW, H2Aub, H2AZ, H3ac, H4ac, in addition to DNA methylation in all three sequence contexts which was presented before) and **genetic features** (sequence composition, presence of Polycomb-responsive elements i.e. PRE motifs, length, superfamily and genomic distribution).
These analyses revealed **significant differences** between LACKs and LOCKs. We have indeed found common features specific of the LOCK (whether they are defined in *drm2* or *rdd*) and common features of the LACKS (whether they are defined in *drm2* or *rdd*) respectively and significant differences between LOCK and LACK. Essentially, LACKS tend to be less DNA methylated than LOCKS with less smallRNA accumulation, less constitutive heterochromatin marks but more facultative heterochromatin marks/hallmarks of stress-responsive genes such as H2Aub, H2AZ, H3/H4ac , and less PREs motifs than LOCKs.

<u>The analyses are presented in Figures 2e-h, Supplementary Figures 2d-i as well as Figures 3e-h and Supplementary Figures 4c-h.</u>

They are described in the revised manuscript as below as indicated below and discussed in the appropriate section.

- Between line 205 and line 218 (meta-analysis of LOCKs and LACKs defined with WT versus *drm2* comparison= DRM2-LOCKs and DRM2-LACKs)

"*Additional meta analyses using published datasets…*
… Together, this points to particular epigenetic and genetic signatures of LACK versus LOCK which likely both contribute to explain the locus-specific and opposite effects of DRM2 on H3K27me3 deposition".

- Between line 258 and line 279 (meta-analysis of LOCK and LACK defined with WT versus *rdd* comparison)

"To further characterize these subsets, we performed meta-analyses …
… The opposing outcomes at these loci are associated with distinct genetic and epigenetic signatures shared across the corresponding DRM2- and RDD-defined classes, and point to locus-specific rules that govern the interplay between DNA methylation dynamics and Polycomb targeting."

- Discussion between line 347 and line 372

"Taken together, our results point to locus-specific rules for H3K27me3 deposition, which would either require DNA methylation or be antagonized by its presence (Fig. 5b). We have named these two classes…
… For instance, it could be predicted that LOCK-specific TF which recognize PREs are sensitive to DNA methylation while LACK-specific TF, associated to different sequence motifs bind to DNA methylation (DRM2-dependent or not based on the nature of the sequence motif).

In addition, to directly test whether the contrasting behaviors are determined by intrinsic properties of Copia sequences—such as sequence composition or the production of small RNAs—we performed the experiment suggested by the reviewer. **We cloned a second Copia element,** COPIA27**, which differs from COPIA21 in both sequence features** (including a higher number of predicted Polycomb-responsive elements) **and siRNA accumulation**. We repeated our transgene assay using these elements. As previously described (Hure et al., 2025), this neo-insertion assay allows us to evaluate the deposition of epigenetic marks independently of genomic position effects.

Strikingly, in contrast with neo-inserted COPIA21, which loses H3K27me3 in *drm2*, the neo-inserted COPIA27 elements gain H3K27me3 in *drm2*. We thank the reviewer for suggesting this experiment, which **indeed revealed a clear functional contrast between two distinct Copia sequences** and further **supports our genome-wide conclusion** that the dual effect of DRM2-mediated DNA methylation on H3K27me3 may be **determined by a combination** of sequence composition, small RNA accumulation, DNA methylation levels, and other epigenetic features associated with individual loci.

We have added these new analyses of de novo DNA methylation and H3K27me3 deposition at the COPIA27 neo-inserted sequence in WT and *drm2* in **Fig. 1a (right panel)**, **Fig. 1d**, **Fig. 1g**, Supplementary **Fig. 1a (right)**, and Supplementary **Fig. 1d**, and describe them in the revised manuscript in lines 151 to 170 and in discussion lines 332-335 (as well as in appropriate places in abstract and introduction).
L.151"*To test if this dependency of H3K27me3 on DRM2-mediated de novo DNA methylation is general of all neo inserted TEs,…)*

L.332". … de novo deposition of H3K27me3 was impaired in the drm2 mutant at a neo-inserted COPIA21 but not at a neo-inserted COPIA27 sequence where H3K27me3 was enhanced, showing a dual effect of de novo DNA methylation on H3K27me3 recruitment…"

Important: In addition to the above, you must comply with the following editorial requests; we will not be able to proceed with your revised manuscript otherwise. Please also see the Nature Communications formatting instructions, which you may find useful while preparing your revised manuscript.

POLICIES AND FORMS REQUIRED FOR RESUBMISSION
* Please complete or update the following checklist(s) to verify compliance with our research ethics and data reporting standards. Address all points on the checklist, revising your manuscript in response to the points if needed.
The form(s) must be downloaded and completed in Adobe Reader rather than opened in a web browser. Each form must be uploaded as a Related Manuscript file at the time of resubmission.

Editorial policy checklist:
https://www.nature.com/documents/nr-editorial-policy-checklist.pdf

Reporting summary:
https://www.nature.com/documents/nr-reporting-summary.pdf

Nature journals have recently announced an update to our guidance on reporting on sex and gender in research studies (see here). We strongly encourage researchers to follow the 'Sex and Gender Equity in Research – SAGER – guidelines' and to include sex and gender considerations for studies involving humans, vertebrate animals and cell lines where relevant to the topic of study (an overview can be found here). Authors should use the terms sex (biological attribute) and gender (shaped by social and cultural circumstances) carefully in order to avoid confusing both terms.

DATA AND CODE AVAILABILITY
* All Nature Communications manuscripts must include a "Data Availability" section after the Methods section but before the References. If any of the data can only be shared on request or are subject to restrictions, please specify the reasons and explain how, when, and by whom the data can be accessed. For more information on this policy and a list of examples, see:
https://www.nature.com/documents/nr-data-availability-statements-data-citations.pdf

Please also include a "Code Availability" section after the "Data Availability" section. If the code can only be shared on request, please specify the reasons. For more information on our code sharing policy and requirements, please see:
https://www.nature.com/nature-portfolio/editorial-policies/reporting-standards#availability-of-computer-code

* As Nature Portfolio policies strongly encourage you to share your research data in a public repository (e.g. spreadsheets, text, images), we are partnering with the figshare repository so that you can use the figshare integration via the 'Research Data Deposition' tab when

submitting your revised manuscript.

Data are stored privately until a manuscript decision is reached and you can edit/withdraw them up to this point: you retain rights and control over your data. The data will be published at the same time as your article; you will receive a data DOI, with guidance on linking the data and manuscript. In the event your manuscript is not accepted, you can keep or remove your data in figshare.

We recommend the use of discipline-specific repositories where available and for a number of data typesthis is mandatory. Ensure you do not submit these data types or any sensitive data to figshare.

* We strongly encourage you to deposit all new data associated with the paper in a persistent repository where they can be freely and enduringly accessed. We recommend submitting the data to discipline-specific and community-recognised repositories; a list of repositories is provided here: http://www.nature.com/sdata/policies/repositories
Refer to our data policies here: https://www.nature.com/nature-portfolio/editorial-policies/reporting-standards#availability-of-data

* To maximise the reproducibility of research data, we ask that you provide a Source Data file containing the raw data underlying the following types of display items:
- Any reported means/averages in box plots, bar charts, and tables
- Dot plots/scatter plots, especially when there are overlapping points
- Line graphs
- Uncropped and unprocessed scans of all blots and gels including all quantified replicates. The edge of membranes, molecular weight ladders and loading controls should be presented on all blots. Where membranes have been cut, please ensure that at least one marker above and below is present. For an example of presentation of full scan blots, see the Source Data file of https://www.nature.com/articles/s41467-020-16984-1#Sec35 and for more information, please refer to https://www.nature.com/nature-research/editorial-policies/image-integrity.

The data should be provided in a single Excel file with data for each figure/table in a separate sheet, or in multiple labelled files within a zipped folder. Name this file or folder 'Source Data', and include a brief description in your cover letter. The "Data Availability" section should also include the statement "Source Data are provided with this paper."
To learn more about our motivation behind this policy, please see: https://www.nature.com/articles/s41467-018-06012-8

A Source Data file is not necessary if all display items presented in the main manuscript and supplementary information can be reproduced from raw data and code that have already been shared in a public repository.

Please replace your bar graphs with plots that feature information about the distribution of the underlying data. All data points should be shown for plots with a sample size less than 10. For larger sample sizes, please consider box-and-whisker or violin plots as alternatives. Measures of centrality, dispersion and/or error bars should be plotted and described in the figure legend.

AUTHOR CHANGES ON REVISION

If there are any changes to the author list in the revised manuscript, please use this approval form www.nature.com/documents/nr-author-list-change-form.pdf, arranging for all authors on your paper to sign the statement confirming that they agree to the author list being changed, and add this document to your resubmission.

HOW TO SUBMIT

Please use the link below to submit the following items as separate documents:
- Revised manuscript
- Any supplementary files
- Point-by-point response to the reviewers' comments, reproduced verbatim
- Cover letter to the editor
- Any completed checklist(s)

https://mts-ncomms.nature.com/cgi-bin/main.plex?el=A7S2EHXb1A3SXTu2I2A9ftdSmoHOvM8KYMhySs8N6DbLgZ

** If you wish to forward this email to your coauthors, please delete the link above **

We hope to receive your revised paper within three months, but we understand that revisions may take longer. Please let us know if you find that the revision process will take substantially more time.

When evaluating your revised manuscript, we will not consider any similar papers published independently in the meantime to compromise the novelty of your study. See here for more information.

Best regards,

Hui Hua, PhD
Senior Editor
Nature Communications

We have complied with Nature communications guidelines, please let us know if there are remaining issues

RESPONSE TO REVIEWERS' COMMENTS

Reviewer #1 (Remarks to the Author):

My concerns have been addressed. I am satisfied with this revision and support publication of this version.

Reviewer #3 (Remarks to the Author):

The authors have addressed my comments very well, bravo. However, by shoe-horning all the new data into the original manuscript, it becomes hard to digest. The summary diagram helps, Figuure 5. But I suggest it should addressed/explained more directly in the discussion for the benefit of the reader, rather than in passing in the discussion. More clarity is needed.

- ➔ **Response:** We thank the reviewer for the positive evaluation of our work and for this helpful suggestion. To improve clarity, we have revised the Discussion to more directly and explicitly describe the model summarized in Figure 5. In particular, we now guide the reader through the different scenarios depicted in panels a and b, clarifying how our experimental results support each aspect of the model and improving the overall flow of the Discussion, which we have also shortened.
