# Supplementary material for: DNA methylation either antagonizes or promotes Polycomb recruitment at transposable elements in Arabidopsis
Source: Nat Commun. 2026 Sep 2;17:10439. doi: 10.1038/s41467-026-77108-9 (PMC13631316; doi:10.1038/s41467-026-77108-9)
Supplement: Supplementary file 4 [file 41467_2026_77108_MOESM4_ESM.pdf]

Description of Additional Supplementary Files

Supplementary Data 1 - AlphaFold-Multimer predictions of putative DRM2–PRC2 interaction interfaces and associated confidence scores.

Supplementary Data 2 - Lists of LOCK (Loci of Opposition between Cytosine methylation and H3K27me3) and LACK (Loci of Association between Cytosine methylation and H3K27me3) identified in *drm2***,** *rdd*, and *met1* mutants.
